# Supercentenarians that Survived COVID-19

**DOI:** 10.14336/AD.2021.0806

**Published:** 2021-10-01

**Authors:** George Siopis

**Affiliations:** ^1^School of Life and Environmental Sciences, The University of Sydney, Sydney, NSW, Australia; ^2^Charles Perkins Centre, The University of Sydney, Sydney, NSW, Australia

**To the Editor**

The novel coronavirus has to date resulted in more than 200 million cases and more than four million deaths around the world. Coronavirus disease 19 (COVID-19) was declared a pandemic by the World Health Organisation on the 11^th^ of March 2020. In what has unfolded as the most concerted effort in global epidemiology, data accumulate on a daily basis to elucidate the risk factors and inform public health initiatives and actions. Age and certain comorbidities have been identified as major risk factors (www.cdc.gov/coronavirus/2019-ncov/need-extra-precautions/older-adults.html). Given that 1 in 10 people across the world are over the age of 65 years, and the increasing ageing trend of the global population, it is important to determine the risk factors within this population too (https://www.un.org/en/development/desa/population/publications/pdf/ageing/WorldPopulationAgeing2019-Highlights.pdf).

At least a quarter of a thousand people over the age of 105 years have tested positive for the severe acute respiratory syndrome coronavirus 2 (SARS-CoV-2) (https://gerontology.wikia.org/wiki/List_of_oldest_people_with_COVID-19). Remarkably, more than half of the completed cases have successfully recovered from the SARS-CoV-2 infection (101 have survived and 97 have died, as of the 3^rd^ of August 2021). It is worth noting that among the people that successfully recovered is the current verified second oldest person in the world, Ms Lucille Randon from France. Ms Randon recovered from SARS-CoV-2 in January 2021, at the age of 117 years (https://www.reuters.com/business/healthcare-pharmaceuticals/europes-oldest-person-117-year-old-french-nun-survives-covid-19-2021-02-09/). Other older adults, have managed to recover from SARS-CoV-2 infections multiple times, with Ms Iris Estay from Chile being the oldest one to recover twice, first in October 2020 and then in March 2021 (https://gerontology.wikia.org/wiki/Iris_Estay).

The ability of some supercentenarians to recover from SARS-CoV-2 infections raises the question whether old age and its associated immunosenescence is less of a risk factor for the outcome of the infection than certain comorbidities. Several epidemiological data have accumulated that shine light on the risk factors for morbidity and mortality with COVID-19. The Centers for Disease Control and Prevention (CDC) has reported that four in five of the people hospitalised in the US with COVID-19 were either overweight or obese (www.cdc.gov/mmwr/volumes/70/wr/mm7010e4.htm?s_cid=mm7010e4_x), and identified obesity as the strongest risk factor for mortality among 540,667 adults [1]. Age-stratified analyses revealed that the number of comorbidities was higher in older adults, with cardiometabolic conditions being the most common in patients of 65 years or older [1]. Although the anthropometric and medical characteristics of supercentenarians surviving SARS-CoV-2 infections are not publicly available, it can be seen from their pictures that they are within a normal weight range and that they do not, phenotypically at least, manifest signs of metabolic syndrome (https://gerontology.wikia.org/wiki/List_of_oldest_people_with_COVID-19). Of course, the absence of obesity does not preclude the presence of comorbidities that are known to accumulate with age, however it can be argued that these individuals would be metabolically healthier than their overweight peers.

The adverse effects of the metabolic syndrome on the immune response have previously been described [2]. Both obesity and diabetes negatively regulate the immune system via causing structural, functional and trafficking changes of leukocytes, and by interfering with the function of the complement and the integrity of the lymphoid tissue [2]. In addition to the protective effects of maintaining a normal weight, physical activity reportedly confers protection against SARS-CoV-2, with both cardiorespiratory fitness and strength reducing the risk of morbidity and mortality from SARS-CoV-2 infection, even many years later in life and independent of adiposity status [3,4].

Unfortunately, there is no publicly available demographic or medical information on the centenarians that survived SARS-CoV-2 to facilitate an insight in regard to the factors that promote a healthy immune system and longevity in the ageing population. It is thus not known if these old survivors were engaging in any form of physical activity either during recent years or during the earlier years of their life, and if so, what form of activity they were participating in. It is also not known if they had any comorbidities and whether these were managed. The only publicly available information is their age and short, unverified biographical notes that can be used to produce some educated, albeit with known limitations, inferences. This perspective attempted to draw conclusions based on such inferences stemming from preliminary data that have known limitations. Future epidemiological efforts are warranted to collect data on relevant anthropometric and metabolic profile indices such as weight, body mass index, waist circumference, lipid profile, blood pressure, glycosylated haemoglobin, as well as important functional indices for the ageing population, such as grip strength, to shine light on the determinants of health in older adults in order to produce informed recommendations to promote health, wellness and longevity in the older age.
